# Social complexity in bees is not sufficient to explain lack of reversions to solitary living over long time scales

**DOI:** 10.1186/1471-2148-7-246

**Published:** 2007-12-21

**Authors:** Luke B Chenoweth, Simon M Tierney, Jaclyn A Smith, Steven JB Cooper, Michael P Schwarz

**Affiliations:** 1 Biological Sciences, Flinders University GPO BOX 2100, Adelaide, SA 5001, Australia; 2 Smithsonian Tropical Research Institute, Apartado Postal 0843-03092, Panama, Republica de Panama; 3 Evolutionary Biology Unit, South Australian Museum, North Terrace, Adelaide, S.A. 5001, Australia

## Abstract

**Background:**

The major lineages of eusocial insects, the ants, termites, stingless bees, honeybees and vespid wasps, all have ancient origins (≥ 65 mya) with no reversions to solitary behaviour. This has prompted the notion of a 'point of no return' whereby the evolutionary elaboration and integration of behavioural, genetic and morphological traits over a very long period of time leads to a situation where reversion to solitary living is no longer an evolutionary option.

**Results:**

We show that in another group of social insects, the allodapine bees, there was a single origin of sociality > 40 mya. We also provide data on the biology of a key allodapine species, *Halterapis nigrinervis*, showing that it is truly social. *H. nigrinervis *was thought to be the only allodapine that was not social, and our findings therefore indicate that there have been no losses of sociality among extant allodapine clades. Allodapine colony sizes rarely exceed 10 females per nest and all females in virtually all species are capable of nesting and reproducing independently, so these bees clearly do not fit the 'point of no return' concept.

**Conclusion:**

We argue that allodapine sociality has been maintained by ecological constraints and the benefits of alloparental care, as opposed to behavioural, genetic or morphological constraints to independent living. Allodapine brood are highly vulnerable to predation because they are progressively reared in an open nest (not in sealed brood cells), which provides potentially large benefits for alloparental care and incentives for reproductives to tolerate potential alloparents. We argue that similar vulnerabilities may also help explain the lack of reversions to solitary living in other taxa with ancient social origins.

## Background

Highly social insect groups have had enormous ecological success [1], yet eusociality has evolved very infrequently [2], raising the question of what barriers there might be to its origin. Furthermore, soldier castes have been lost in some thrips [3] and aphids [4], resulting in the loss of eusocial nesting strategies. In halictine bees there have been three origins of sociality but as many as twelve losses, suggesting that in an evolutionary sense complex sociality may be difficult to gain, but easy to lose [5]. In contrast, there have been no losses of sociality in the ants, termites, vespid wasps and corbiculate bees, all of which evolved sociality > 65 mya [6].

In both thrips and halictid bees, sociality has evolved much more recently than the Cretaceous origins of sociality in ants, termites, corbiculate bees and vespid wasps. McLeish *et al*. [7] showed that sociality in gall forming thrips evolved less than 10 mya, and Brady *et al*. [6] showed that the three origins of sociality in halictines are also relatively recent (22 – 20 myBP). This raises the question of whether losses of sociality are more likely in lineages where sociality is relatively recent, compared to older social lineages that may have reached a 'point of no return'.

The notion of a 'point of no return' was first suggested in the early 1970s by Wilson [1] and proposes that, given suitable evolutionary time, the multiple and integrated adaptations associated with highly complex social behavior may preclude reversions to less complex or non-social life cycles. Wilson and Hölldobler ([8], p. 13368) refer to this evolutionary point as one where it is either "impossible, or at least difficult and uncommon", for a eusocial species to revert back to a more primitively social or non-social form of organization, and speculate that it coincides with the evolution of an anatomically distinct worker caste. The conjecture is important because it proposes a degree of irreversibility in social evolution due to integration among adaptations in multiple traits, a situation akin to the idea of phylogenetic inertia arising from bauplan constraints [9].

Importantly, the 'point of no return' hypothesis seems to be the only one proposed for the lack of reversions to solitary life-cycles in the major social insect groups and as such is an almost default paradigm. This is surprising, given the amount of debate on other aspects of social evolution. Yet the point of no return hypothesis lacks a clearly stated underlying mechanism for why reversions are precluded and, as such, is not truly falsifiable. Nevertheless it is open to indirect assessments: in particular, demonstrating that very long term maintenance of sociality does not require social complexity or a distinct worker caste would indicate the possibility of alternative explanations for long term maintenance of sociality.

Indirectly assessing whether points of no return depend on social complexity and distinct worker castes can be achieved by examining patterns of origins and losses in taxa where these two traits are absent or variable. Brady *et al*.'s [6] study of origins and losses in halictine bees provides one such assessment. Halictine bees provide special insights into social evolution because, unlike most other eusocial groups, adult females in all species are totipotent and capable of producing brood [10]. Thus, individual females are not constrained to group nesting, so that evolution is able to 'explore' non-social options. Only one other group of bees, tribe Allodapini (Apidae), is speciose, exhibits diverse range in forms of sociality and, in virtually all species, all females are totipotent [11].

Until recently it was thought that sociality in allodapines had arisen comparatively recently among the extant lineages [11] from a subsocial ancestor (i.e. a solitary ancestor displaying extended parental care), and this ancestral trait had been retained in a phylogenetically basal African allodapine, *Halterapis nigrinervis *[12]. At the same time, nesting biology of the speciose *Halterapis *group from Madagascar was unknown. Recent molecular analyses [13] show that the African *Halterapis *is not basally situated in the allodapine phylogeny, and that the African and Malagasy members of this genus are in fact paraphyletic, implying a need for future taxonomic revision. Using both Bayesian and penalized likelihood approaches, Schwarz *et al*. [14] indicated an origin of the allodapine tribe as being >40 mya but pointed out that this is likely to be a highly conservative estimate. Recent behavioral studies also show that the Malagasy *Halterapis *display complex social behavior [15,16]. These findings indicate that sociality is plesiomorphic for the allodapines, and despite the relatively ancient origin of the tribe, the African *Halterapis *is the only allodapine to have potentially lost complex social behavior.

## Results

The initial study of *H. nigrinervis *[12] was based on a sample size of only eleven nests and multifemale colonies comprised a single inseminated female along with one to several uninseminated females that were presumed to be recently eclosed adults and soon to disperse.

**Figure 1:**
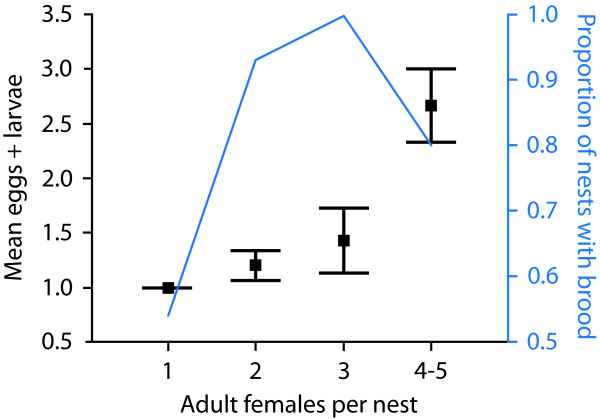
**Variation in the number of eggs + larvae among different colony sizes**. Brood numbers were 1-truncated (colonies lacking any eggs or larvae were excluded). A Kruskal Wallis non-parametric test indicated a significant effect of the number of adult females on the number of eggs and larvae present within nests (χ^2^_3 _= 12.145, P = 0.007). The blue line displays the proportion of nests containing brood for different colony sizes.

**Figure 2:**
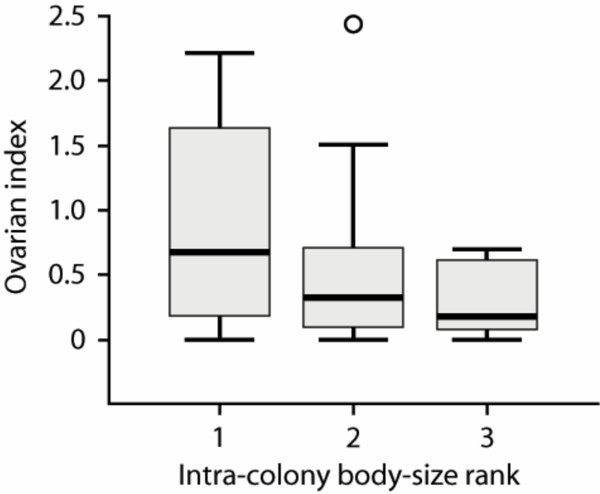
**Mean ovarian index versus intra-colony body size rank**. Mean ovarian index (± 1 S. E.) as a function of intra-colony body size rank for adult females from multifemale colonies of *H. nigrinervis*. Individuals with identical sizes were given the same sequential rank. By regressing the ovarian index of individuals within a nest on their residual body size, we found a significant decrease in ovarian index as relative female body size decreases within colonies (P = 0.008).

Our sample comprised a total of 52 *H. nigrinervis *nests. Seven nests were stored in ethanol and the remaining 45 in Kahle's solution for dissection. These samples (Table 1) indicate that *H. nigrinervis *is indeed social because: (i) approximately half (54%) of the nests collected were multifemale; (ii) multifemale nests were far more likely to contain brood (Table A1, Additional File 1) and the number of young brood (eggs and larvae) increased with the number of adult females, indicating enhanced brood production when multiple adult females are present within a nest (Figure 1); (iii) sex ratios were strongly female-biased, consistent with positive kin interactions leading to local resource enhancement [17,10]; and (iv) ovary sizes of nestmates were strongly influenced by intra-colony body-size rank (Figure 2) indicating the existence of reproductive hierarchies. These results mean that *H. nigrinervis *is social and that there are consequently now no known losses of sociality in allodapines.

**Table 1:** Colony contents data. Brood numbers and sex ratio parameters for 52 nests of *H. nigrinervis *collected from Grahamstown, South Africa in late summer (February).

Adult females/nest	Per capita eggs+larvae	Mean total brood (eggs, larvae prepupae and pupae)	Mean sex ratio of pupae (total male pupae: total female pupae)	Number of nests
1	0.292	0.75	0 (0:2, 2 nests)	24
2	0.60	2.13	0.22 (2:7, 9 nests)	15
3	0.503	2.50	0 (0:5, 5 nests)	8
4	0.313	3.50	0.33 (2:4, 3 nests)	4
5	0.60	3.00	-(0 nests)	1

Consensus phylogenies from three partitioned Bayesian analyses of sequence data all had identical topologies and almost identical branch lengths. The consensus cladogram with posterior support values is given in Figure A1 (Additional File 1). Ingroup generic-level bifurcations were highly supported and consistent with other sequence-based studies of allodapines [14]. We also analysed sequence data with a maximum parsimony approach and that gave broadly consistent results to our Bayesian analyses and previous phylogenetic studies [14] (Figure A2, Additional File 1). The only inconsistencies between our Bayesian, our MP results, and those of previous studies, involve nodes close to terminal taxa.

**Figure 3:**
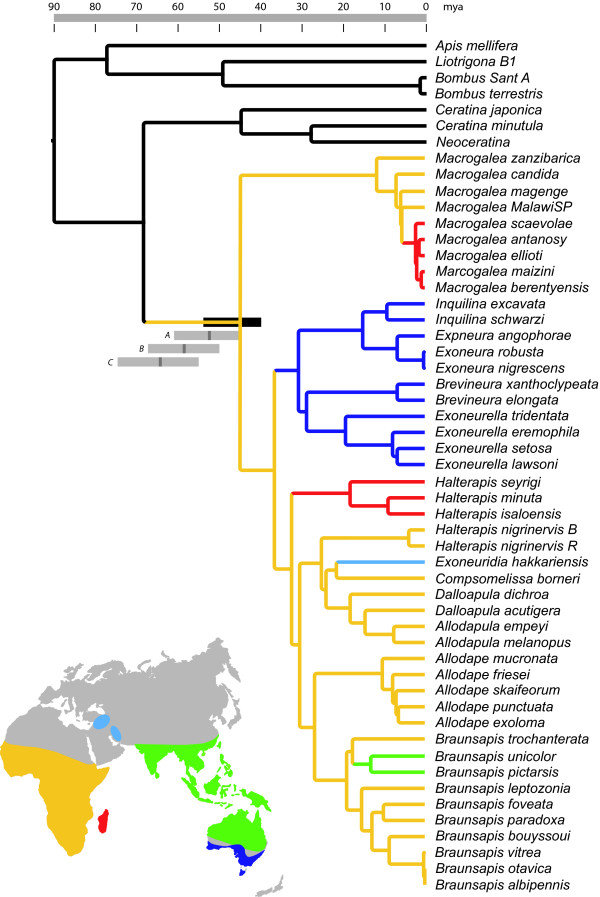
**Chronogram of the allodapines derived from penalized likelihood transformation of a consensus Bayesian phylogram**. 95% central distribution limits for the allodapine root node are indicated by the black bar, assuming a 90 myBP divergence between the xylocopine and corbiculate lineages, and by grey bars assuming divergence times of 100 (bar A), 110 (B) and 120 (C) myBP. Geographic distributions of clades are colour coded according to the map.

We used penalized likelihood transformation of the Bayesian phylograms to produce a chronogram (Figure 3), which also indicates the geographic distribution of the major clades. When the basal node (divergence of corbiculate bees from the xylocopine bees) is conservatively set at 90 myBP, the point estimate for the earliest divergence of allodapine clades is 47 myBP, with a lower (most recent) 95% limit of 40 myBP. Because sociality occurs in all the extant allodapine lineages, it must be a plesiomorphic trait and must therefore have originated by at least 40 myBP, and probably much earlier given the very conservative nature of all three calibration points. This age-estimate is similar to that of Schwarz *et al*. [14] and suggests that the age of the allodapine root node is approximately twice the ages for estimated origins of sociality in halictine bees [6].

As an independent assessment of approximate divergence ages, we compared pairwise maximum likelihood distances for allodapine species whose most recent common ancestor (MRCA) was at the root allodapine node with those for halictine species whose MRCA was at one of the three origins of sociality in halictines [6], which were dated at 20–22 mya [6], using a sequenced fragment of EF-1α common to both groups. Substitutional parameters for this gene fragment are almost identical for halictines and allodapines (see supplementary material) indicating that evolutionary dynamics for this gene fragment are very similar for the two bee groups. If evolutionary rates are also comparable in the two groups, the resulting distances suggest that the root node of the allodapines is about twice the age of the origins of sociality suggested for the halictines (Figure A3, Additional File 1), which concords very closely with our penalised likelihood analysis.

## Discussion

Our results are important because they suggest that, compared to halictine bees, allodapines have a much older origin of sociality but show no losses of sociality. In this respect, they are more similar to eusocial lineages such as ants, corbiculate bees, vespid wasps and termites, which also have ancient social origins with no losses. This raises the question of what factors may prevent losses of sociality over very large time scales.

Lack of reversions to solitary living in allodapines cannot be explained by arguments that they are restricted to habitats or climatic regimes that favour sociality. The ecological diversification of allodapines covers habitats such arid gibber deserts, savannas, bushvelds, coastal heathlands, equatorial and subtropical rainforests, and sub-alpine regions [12,18]. Colony phenology ranges from highly seasonal univoltine egg production schedules [18], all the way through to asynchronous development patterns where egg production, brood maturation, and foundress dispersal occur year round [10]. The lack of reversions to purely solitary living is even more notable because of the near-ubiquity of female totipotency in allodapines [10], which means that physiological factors do not preclude independent living. We argue that the absence of reversions to solitary living is due to ecological consequences of the way in which allodapines rear their brood, and this may also help explain some broader trends found in ants, termites and vespid wasps.

Unlike virtually all other bees, which rear their brood in fully provisioned sealed cells, allodapines rear brood in un-partitioned and unsealed tunnels. Because brood lack the physical protection of an enclosed cell, they are highly vulnerable to predation in the absence of an adult guard. Indeed, the major benefit of group living in allodapines is avoidance of total brood failure [18], and this benefit is greatest when comparing single- with two-female colonies, because brood in the former colonies are unprotected when the sole adult is foraging [18]. This vulnerability of brood is heightened by orphaning, since not only will post-feeding brood be unprotected, but feeding-stage larvae will be unable to complete their development due to lack of food. In the event of orphaning, potential alloparents could reap large indirect fitness gains by simply protecting post-feeding brood or completing the feeding of partially reared larvae [19], as well as direct benefits from inheriting a nest along with a subsequent cohort of potential alloparents. In fact, alloparental rearing in the absence of possible mothers is recorded from diverse species [20,21] indicating that it does not require maternal coercion. At the same time, mothers would gain benefits from permitting some daughters to remain in the nest as insurance against orphaning [22].

In contrast, the vulnerability of brood and the benefits of alloparental care in cell-provisioning insects are quite different. Mass provisioning of brood in cells means that mothers sequester their investments as completed units over time, and these cells provide brood with some physical protection from enemies at the nest. In addition, if orphaning does occur, immatures sealed in cells will have sufficient food for their complete development, further reducing the scope for benefits from alloparental care.

Whilst protecting the nest from both predation and parasitism is important for all bees [11,23], allodapine brood are particularly vulnerable due to the lack of cells [10,11]. Continuous protection from predation is impossible for a solitary nesting allodapine, as the nest is completely unguarded during the female's foraging trips. We suggest this vulnerability is a compelling explanation for why there have been no reversions to purely solitary living in allodapines but multiple losses in halictines.

It has been argued that the lack of reversions to solitary nesting in ants and termites may reflect the evolution of social complexity to a 'point of no return' [8], where a species is no longer able to live solitarily. However, there are numerous ant groups where newly founded colonies involve non-claustral queens rearing their first brood cohort to maturity without help from workers [24], suggesting that competency for solitary brood rearing *per se *exists in many taxa. For example, within many ant subfamilies (predominantly within in the poneroid clade but also some formicoid subfamilies [24,25]), there are species where colony foundation almost exclusively involves a single brood-rearing foundress and very small ultimate colony sizes [24,26]. Queens must forage in order to feed their first clutch of brood, indicating that solitary-founding females in these species are capable of foraging effectively enough to rear through their first generation of brood alone [26]. Many of the aforementioned subfamilies also contain multiple independent losses of the queen caste, which has resulted in numerous species displaying ubiquitous female totipotency [26] but without any transitions to purely solitary living.

In social taxa where females can successfully rear brood to maturity in a non-claustral fashion without help of a worker caste, explanations for a lack of reversions to solitary living must involve something other than incompetence for independent brood rearing. Ants share one key life history trait with allodapines, namely that brood are progressively reared in unsealed communal tunnels. In social vespid wasps, larvae are also reared to pupation in unsealed cells, and while termite young are not 'provisioned' by adults, they also develop in unsealed chambers and are highly vulnerable in the absence of adults. In all these taxa, protection of brood depends heavily on adults, and this contrasts with halictines where nearly all species sequester their fully provisioned brood in closed cells. Losses of sociality would remove the brood protection that group living confers in the former groups, but the physical protection of cells in halictines would allow some protection of brood in solitary nesting halictines to be maintained.

Lastly, we have argued that the 'point of no return' paradigm has been framed in a way that does not readily allow falsification. We believe that our conjecture provides two predictions that permit empirical assessment: (i) that reversions from social to solitary brood rearing are more likely in clades where constraints to solitary living are low; and (ii) that reversions from social to solitary can occur even after specialised worker/soldier castes have evolved, provided that ecological constraints for independent reproduction are relaxed. Halictine bees and gall-forming thrips comprise two groups where these predictions could be tested, but there are likely to be many other taxa as well.

## Conclusion

Our findings suggest a very different framework for understanding social evolution from that argued in some recent studies [27,28] that emphasize the importance of mechanistic approaches involving physiology, regulatory circuits and genetic-networks. In particular, Hunt and Amdam [29] suggest "...that social evolution in insects can be fully – and finally – understood" by such mechanistic approaches. Although such approaches may help explain the ontogeny of worker-like behaviour, our results indicate that retention of such behaviour is likely to be due to ecological and life-history factors, and these have the potential to determine very long term patterns of social evolution. The ecological dimensions of sociality cannot be ignored when trying to understand its origins and long-term maintenance.

## Methods

### Sociality

Colonies of *H. nigrinervis *were collected from Grahamstown, South Africa in 2005 from 20 to 23 February, when colonies were rearing brood. Colonies were preserved in ethanol for molecular studies and Kahle's solution for dissections. Ovarian indices of females were calculated as the summed lengths of the three largest oocytes divided by wing length (used as an indication of body size), and used when estimating ovarian enlargement to avoid body-size scaling effects. Colony productivity was measured as the number of eggs and larvae divided by the number of adult females.

### Benefits of social nesting

If multifemale colonies contain only a single mother and other females are recently eclosed daughters who are soon to disperse, we would expect that the latter daughters would not have an effect on the number of brood being actively reared (larvae) or soon to be reared (eggs). We examined this statistically. Variation in the number of eggs + larvae among different colony sizes was assessed using Kruskal Wallis non-parametric test, rather than parametric ANOVA, because brood numbers were 1-truncated (colonies lacking any eggs or larvae were excluded). This test indicated a significant effect (χ^2^_3 _= 12.145, P = 0.007) and Figure 1 below indicates an increasing function.

### Molecular phylogenetics

Using molecular data, Schwarz *et al*. [14] argued that the Allodapini had an origin in the Eocene of at least 40 mya. Here, we re-examine the time of this origin using penalised likelihood transformation of a molecular phylogeny based on an expanded set of taxa that allows an additional fossil calibration point and better resolution of some internal nodes including the position of *Halterapis*. We also explore the effects of varying the date of the basal node, namely the divergence of lineages leading to the allodapine and corbiculate bee clades.

We used two mitochondrial (COI and cyt b) gene regions and an exon region of one nuclear gene (F2 copy of EF-1α) comprising 1279, 428 and 772 nucleotides respectively. Our taxa comprised at least two species of each non-parasitic allodapine genus except *Exoneuridia *and *Compsomelissa *which are rare and for which we had only one species each. Most of the ceratinine and allodapine taxa used in our study have been used in previous phylogenetic analyses of allodapines by Schwarz *et al*. [13,30,15,14] and Bull *et al*. [31] and GenBank accession numbers are provided in these manuscripts. In addition to these taxa, we used a Malagasy *Halterapis*, *Halterapis isaloensis *(EU254247, EU254248, EU254249) and an additional African *Halterapis*, *Halterapis nigrinervis *with a black metasoma (EU254250, EU254251). We used two halictid bees, *Lasioglossum lanarium *and *Agapostemon tyleri *[32] as the outgroup. *Apis mellifera*, as well as representatives of the genus *Bombus *(*Bombus terrrestris *and an unidentified *Bombus sp*. from Santiago, Chile (EU254244, EU254245, EU254246)) and an unidentifiable species of *Liotrigona *from Madagascar (EU254241, EU254242, EU254243) were included to allow additional calibration points for determining the age of the allodapine root node (see below).

DNA extraction, amplification and sequencing methods used for the gene fragments used on our analyses have already been published, see Schwarz *et al*. [14] and references cited therein.

Phylogeny was inferred using both Maximum parsimony and Bayesian inference. We place greater reliance on Bayesian methods for allodapines because of greater ability of this approach to deal with heterogeneity in substitutional dynamics of different codon positions and gene fragments, as well as problems arising from signal degradation at third codon positions [32]. Bayesian inference was implemented with MrBayes v3.0.4 [33], with codon positions separately partitioned for the nuclear and combined mitochondrial genes, and all partitions unlinked for model parameters and using default priors, as described by Schwarz *et al*. [14]. Three Bayesian runs were undertaken in order to ensure runs were consistently converging on similar patterns. MCMC chains were run for 3 × 10^6^generations, with a burnin of 1.5 million generations, and post-burnin trees combined across runs. Trees were sampled every 500th generation. All runs converged on identical consensus topologies and posterior probability values for nodes and branch lengths were based on a total of 9000 post-burnin sampled trees. The resulting consensus phylogram was transformed into a chronogram using Sanderson's [34] penalised likelihood method. Bayesian methods using relaxed-clock models were not used because the estimated transition matrix clearly contradicted the most complex (F84) model that can be implemented using Thorne and Kishino's [35] Multidivtime software.

For our maximum parsimony (MP) analyses, we ran a heuristic search with 50 random sequence stepwise additions, with 5 trees held at each step, and TBR branch swapping with 500 bootstrap pseudoreplicates to estimate support. 3^rd ^mitochondrial positions were removed from the analysis to prevent long-branch attraction between genera observed in previous studies [14,30,32].

To estimate the age of the allodapine root node (earliest divergence among extant clades) we employed three calibration points. We set a minimum divergence between Ceratinini and Allodapini at 45 myBP because of the existence of fossil Boreallodapini species from Baltic amber [36]. Boreallodapini is the extinct sister group to the extant tribe Allodapini and Ceratinini is the next-most basal tribe. This minimum age is therefore highly conservative [14] and the Allodapini+Boreallodapini is likely to have diverged from the Ceratinini much earlier than this. We also used the presence of the amber fossil stingless bee *Cretotrigona prisca*, dated at 70–65 myBP [37] to set a minimum time of divergence of 70 myBP between the Meliponini and Bombini, but as with the boreallodapine calibration point this is likely to be highly conservative, requiring that *C. prisca *had arisen as soon as the two clades diverged. The last calibration point was the node joining the xylocopine and the corbiculate lineages in our sample. Fossils of the plant family Clusiaceae, whose floral morphology is closely linked to corbiculate bees, are dated at 90 myBP [38] and so we fixed this node at 90 myBP. However, it is possible that corbiculates diverged from the clade leading to xylocopines well before this, so we also explored the effect of setting the date of this node to 100, 110 and 120 myBP. Confidence intervals for node ages were estimated as 95% central distribution limits using the method of Schwarz *et al*. [14], where all post-burnin Bayesian trees were filtered by the consensus Bayesian topology, then subjected to penalised likelihood transformation. The resulting chronograms were sorted for age of the allodapine root node and the resulting 2.5% upper and lower node ages removed.

As a further check of the antiquity of the allodapine root node we compared sequence divergences of EF-1α for species-pairs whose most recent common ancestor occurred at that node, with equivalent species-pairs for the origins of sociality in halictines (three origins, with estimated ages of 22–20 mya) where internal fossil calibration points are available [6,14], with an overlapping region of exon 772 bp. For both data sets, gene fragments were trimmed to the overlapping region and ModelTest 3.0.4 [39] was used to fit substitution models to both taxa. These analyses returned highly similar models (Table A2, Additional File 1), suggesting that this gene region evolves with very similar dynamics in both halictines and allodapines. We then used these models to calculate maximum likelihood pairwise distances for both data sets. For allodapines, this involved pairwise distances between the *Macrogalea *species (forming the sister clade to all other allodapines) and representatives of all other known allodapine non-parasitic genera. For halictines, this involved all taxa pairs whose most recent common ancestor (MRCA) was the inferred node of origin of sociality. These pairwise distances are not independent of each other, since most taxa are included in the calculation of more than one pairwise distance in either bee group, so that mean and median values cannot be statistically compared between the two taxa.

## Authors' contributions

LBC helped conceive the study, carried out the field work, was responsible for all work on sociality and sex allocation in *Halterapis*, obtained DNA sequence data, analyzed the data and prepared the manuscript. SMT contributed very substantial DNA sequence material and help with analyses. JAS helped with fieldwork and DNA sequencing. SJBC oversaw DNA work and helped conceive the study. MPS supervised and helped conceive the study as part of a broader program on allodapine social evolution and phylogenetics and helped with fieldwork and analyses. All authors contributed to interpretation of data and manuscript revisions.

## Supplementary Material

Additional file 1 **Chenoweth *et al***. MS Word document containing additional methodology employed in this study as well as additional figures and tables. Click here for file
