# Supplementary material for: Social complexity in bees is not sufficient to explain lack of reversions to solitary living over long time scales
Source: BMC Evol Biol. 2007 Dec 21;7:246. doi: 10.1186/1471-2148-7-246 (PMC2231370; doi:10.1186/1471-2148-7-246)

**Additional Files –Chenoweth et al.**

**Reproductive hierarchies**

The reproductive hierarchies suggested in Figure 2 (main article) for colonies with three or more females cannot be assessed using regression or correlation analyses because the independent variable is ranked body-size and ranks within a nest are not independent. Therefore, we calculated the residual body size for each female within a nest and then regressed both ovarian index and ovary size on the residuals separately for each nest. If ovary size (or ovarian index) is independent of within-nest relative body size, regression slopes should have equal likelihood of being positive or negative, and deviations from this were tested using a binomial test. Colonies where all females were tied for body size were excluded. The null hypothesis was that there would be an equal likelihood of positive and negative relationships. However, we found that all 12 regression slopes for ovary size were positive, 11 of the 12 coefficients for ovarian index were positive, with a two-tailed significances of P < 0.001 and P = 0.008 respectively. The positive slopes indicate that larger females (within colonies) tended to have larger ovaries.

**Benefits of social nesting**

Previous studies on allodapines have shown that a major benefit of social nesting is prevention of total brood loss in multifemale colonies. We examined this using a log likelihood test with presence/absence of brood crossed with the number of adult females per nest (ranging from 1 – 5, with 4 and 5-female colonies combined because of small samples sizes, Table A1 below). This test indicated significant dependence (χ^2^_3_ = 11.899, P = 0.008).

**DNA sequence distances**

We used sequence divergences of the nuclear gene EF-1α to assess whether the allodapine root node (minimum origin for sociality in allodapines) was likely to be older than the three origins of sociality in halictines. Halictine sequences were obtained from Genbank (accession numbers in S1 and references therein). Pairwise distances were estimated using a maximum likelihood distance, and parameters for this were based on recommended parameters from ModelTest 3.0.4 [S2] using only that part of the gene fragment that was common to both the allodapine and halictine samples, comprising 778 basepairs and excluding the intron in this region, which could not be aligned for most taxa. Evolutionary substitutional parameters were estimated by fitting sequence data to best-known trees for both groups: for halictines this was the topology given by Brady et al. [S1] and for allodapines it was the topology given in this paper (Table A2). A boxplot, giving medians and inter-quartile ranges for all taxa-pairs whose most recent ancestor is the inferred origin of sociality, are presented in Figure A3 below for both allodapines and halictines.

Pairwise distance estimates include the effects of divergences in both lineages descending from the most recent common ancestor to the two species being compared. If the amount of genetic change, modelled by the ML distance parameters, has a linear relationship to time, then relative ML distances for any pair of taxa should be proportional to (time since divergence)/2. That means that if distances are linearly related to time since divergence, then a four-fold difference in distances between two taxa-pairs should correspond, approximately, to a two-fold difference in the divergence ages of the two pairs of taxa being compared.

References

S1. Brady SG, Sipes S, Pearson A, Danforth BN: **Recent and simultaneous origins of eusociality in halictid bees.** *Proc R Soc B* 2006, **273(54):** 1643-1649.

S2. Posada D, Crandall KA: **MODELTEST: Testing the model of DNA substitution.** *Bioinformatics* 1998, **14:** 817-818.

**Table A1 - Number of colonies with or without brood for different sized colonies**

4 & 5- female colonies were pooled because of small sample sizes. Nests containing more than one female were far more likely to contain brood.

|  | Adult females/nest | | | |
| --- | --- | --- | --- | --- |
|  | 1 | 2 | 3 | 4 - 5 |
| Brood absent | 13 | 1 | 0 | 1 |
| Brood present | 11 | 14 | 8 | 4 |

**Table A2 - Substitution parameter matrices for allodapines and halictines**

Parameter matrices for allodapines are based on the data and topology

presented in this paper, and halictine parameter matrices are based on data and topology presented in Brady et al. [S1]. Parameters estimated with ModelTest 3.0.4 comprising base frequencies, substitution rates, proportion of invariant sites and α shapes.

| Model parameters | Allodapines | Halictines |
| --- | --- | --- |
| Base frequencies |  |  |
| A | 0.2604 | 0.2675 |
| C | 0.2079 | 0.1997 |
| G | 0.2456 | 0.2419 |
| T | 0.2861 | 0.2909 |
| Rate matrix values |  |  |
| A-C | 1 | 1 |
| A-G | 5.7094 | 6.0159 |
| A-T | 1 | 1 |
| C-G | 1 | 1 |
| C-T | 11.9618 | 12.2367 |
| G-T | 1 | 1 |
| Prop(invariant sites) | 0.6256 | 0.6126 |
| Gamma shape | 1.3651 | 1.2578 |

**Figure A1 - Consensus cladogram of representatives of major allodapine genera.** Posterior probability values are provided as percentages above nodes. Taxa from the genus *Braunsapis* are abbreviated into the genus name “*Br*”.

**Figure A2 - Bootstrap tree from maximum parsimony analysis of EF-1 α and 1nt and 2^nd^ mitochondrial nucleotides of COI and Cyt *b*.** Bootstrap support values are provided as percentages above nodes. Taxa from the genus *Braunsapis* are abbreviated into the genus name “*Br*”

**Figure A3 - Boxplot of pairwise maximum likelihood distances, calculated using PAUP with the parameters given in Table 3.**

Allodapine distances are for pairwise taxa whose most recent common ancestor is the root allodapine node which is the minimal point for the inferred origin of sociality in allodapines. The same protocol is used for calculating halictine pairwise distances except that there are three inferred origins.


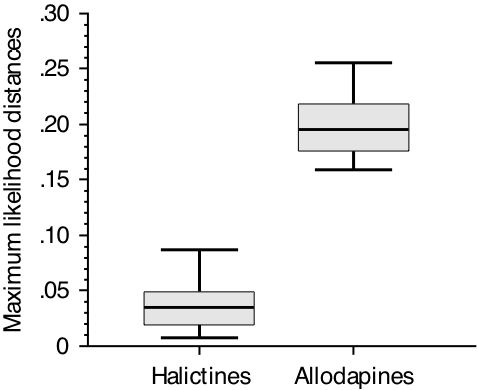

Supplement: Additional file 1 — Chenoweth et al. MS Word document containing additional methodology employed in this study as well as additional figures and tables. [file 1471-2148-7-246-S1.doc]
